# Does Production of *Clarias gariepinus* × *Heterobranchus longifilis* Hybrids Influence Quality Attributes of Fillets?

**DOI:** 10.3390/foods11142074

**Published:** 2022-07-12

**Authors:** Małgorzata Sobczak, Remigiusz Panicz, Jacek Sadowski, Mirosław Półgęsek, Joanna Żochowska-Kujawska

**Affiliations:** 1 Department of Meat Sciences, Faculty of Food Sciences and Fisheries, West Pomeranian University of Technology in Szczecin, 4 Kazimierza Królewicza Street, 71-550 Szczecin, Poland; rpanicz@zut.edu.pl (R.P.); joanna.zochowska-kujawska@zut.edu.pl (J.Ż.-K.); 2 Department of Aquatic Bioengineering and Aquaculture, Faculty of Food Sciences and Fisheries, West Pomeranian University of Technology in Szczecin, 4 Kazimierza Królewicza Street, 71-550 Szczecin, Poland; jacek.sadowski@zut.edu.pl (J.S.); miroslaw.polgesek@zut.edu.pl (M.P.)

**Keywords:** fillet yield, meat, nutritional value, sensory traits

## Abstract

The increase in fish and seafood consumption observed in recent years is a consequence of the growing consumers’ awareness of proper nutrition. One of the possibilities to provide fish-friendly, qualitatively attractive (both in terms of nutrition and sensory quality) raw fish materials is the production of hybrids, which would improve production rates without compromising their meat quality. This study compares the carcass and fillet yields of *Clarias gariepinus* (C) and heteroclarias *C. gariepinus* × *Heterobranchus longifilis* (H), and the nutritional (chemical composition, fat quality) and culinary quality (structure, texture, color, cooking loss, sensory characteristics) of their meat. Significantly (*p* ≤ 0.05) higher carcass and fillet yield was shown for H, whose fillets had comparable sensory quality and proximal composition to C. The meat of H can be an attractive raw material for more demanding groups of consumers—for children and adolescents (due to the less intense fish tastiness), as well as for older people who have problems with chewing and digesting food (due to lower sensory chewiness and perception of connective tissue).

## 1. Introduction

In global aquaculture, the production of interspecific hybrids within the Siluridae family has been successfully carried out for many years. Hybrids often combine the features of parental lines that are desired by breeders, i.e., rapid growth rate, low FCR (feed conversion rate), increased tolerance to low concentration of dissolved oxygen in water, or higher resistance to pathogens (e.g., *Edwardsiella ictaluri*). The most known hybrid in this group of animals is channel x blue catfish (*I. punctatus* (Rafinesque, 1818) × *I. furcatus* (Lesueur, 1840)), in which traits exceed even those observed in individuals of the pure channel catfish line [1]. Another well-known intergeneric hybrid is crossbred between the *Heterobranchus longifilis* (Valenciennes, 1840) and *Clarias gariepinus* (Burchell, 1822), the so-called “heteroclarias”. This hybrid is becoming popular because the fish show very fast growth, inherited from the species *H. longifilis* [2]. In semi-intensive systems, fry of hybrids (mean weight 7.5 g) within 24 weeks reached an average harvest weight of 880 g [3]. In addition, heteroclarias has a higher capacity to grow in unfavorable farming conditions, efficiently utilizes diverse feeds, and has increased disease resistance. The unique breeding features of the *C. gariepinus* × *H. longifilis* hybrid have increased the interest in global aquaculture, which is associated with the development of fish processors interested in new ways to use this raw material. The literature data show that consumers highly rate the quality of meat of *C. gariepinus* [4,5] due to the high content of protein (17.35%) and low calorific value (491 kJ). The meat of this species has an intense red color and a low free leakage, is tender, boneless, and devoid of intense fishy flavor. These factors mean that African catfish meat is characterized by high culinary and processing suitability [5]. Due to its inherent characteristics, catfish meat can gain the interest of new consumers currently not interested in the consumption of fish. The aversion to the taste, smell, and texture of fish, as well as the fear of ingesting bones contribute to low fish intake [6,7]. However, no information was found in the available literature on the quality characteristics of *H. longifilis* meat. It is, therefore, difficult to estimate the effect of these “parental” species on the culinary and processing quality of heteroclarias meat (hybrids of *C. gariepinus* × *H. longifilis*). Moreover, in the scientific literature, there is a low number of data available for heteroclarias, and they are mostly related to the zootechnical parameters. Therefore, it seems that determining the culinary and processing qualities of the new raw meat material will contribute its proper use.

Catfish and its hybrids are important worldwide. The total production of African catfish officially reported by FAO is 246,476 t during 2015 [8]; however, it is expected that the production will increase in the following years. Therefore, detailed characteristics of catfish and its hybrids’ meat is of utmost importance to aquaculture and fish processing sectors in those countries in which catfish production is very popular, such as Nigeria, Netherlands, Brazil, and Hungary. This approach is also in line with the Sustainable Development Goals (SDGs) set in 2015 by the United Nations which indicate the need of sustainable food production. [9]. The aim of the study was to assess selected parameters of nutritional and culinary quality of heteroclarias hybrid meat obtained from crossing female *C. gariepinus* with male *H. longifilis*. The preliminary results obtained in our study may help the fish sector to introduce new raw materials and products on the market that are sensorily appealing to consumers who have to eat seafood products to maintain healthy growth and development, i.e., children and seniors.

## 2. Materials and Methods

### 2.1. Fish, Rearing, and Housing Facilities

This study was carried out at the Department of Aquatic Bioengineering and Aquaculture, Faculty of Food Science and Fisheries, West Pomeranian University of Technology, Szczecin, Poland. Adult *C. gariepinus* and heteroclarias were obtained from the Experimental Fisheries Station in Nowe Czarnowo (53°12′36′′ N, 14°27′48′′ E). Fish from each group were placed randomly in two RAS installations which consisted of 3 tanks (1 m^3^), 3 hydroponic trays (0.5 m^3^), and mechanical and biological filtration. Each tank was stocked with 100 fish of *C. gariepinus* and heteroclarias with an average initial body weight of 247 ± 30 g and 237 ± 25 g, respectively. Fish from each tank were weighted every 14 days. RAS water was refilled when needed and aeration was continuously provided using an air blower. Ambient temperature was 23 ± 2 °C. Lighting in the culture unit was set at a 12:12 light:dark cycle using fluorescent lamps. Water quality parameters, including dissolved oxygen (O_2_), pH, and total ammonia (NH_4_), were monitored on a daily basis. The average value of these parameters throughout the study were 2.5 (±1.0) mg L^−1^, 6.5–7.2, and 0.6–1.2 mg L^−1^, respectively. The fish were fed the 4.5 mm Bona Float (Aller Aqua, Poland) for 7 months (October 2016–May 2017), calculated relatively to the metabolic fish weight. At the end of the feeding trial, 20 fish (10 male and 10 female) of each group were anesthetized with an overdose of 2-phenoxyethanol (Sigma-Aldrich, Taufkirchen, Germany), and body (BW), carcass (CW), and skinned fillet (FW) weights recorded. Carcass yield and fillets yield were calculated using the fallowing equations.

(1) $$\mathrm{Carcass}\;\mathrm{yield}\;\left(\%\right)=\frac{\mathrm{CW}}{\mathrm{BW}}\times 100\%,$$

(2) $$\mathrm{Fillets}\;\mathrm{yield}\;\left(\%\right)=\frac{2\times \mathrm{FW}}{\mathrm{BW}}\times 100\%.$$

The study was conducted according to the “Guidelines for the treatment of animals in behavioural research and teaching” published in Animal Behaviour [10].

### 2.2. pH Measurement

The pH was measured along the longest axis of a carcass, 4 times for each raw fillet. The measurement was performed using a portable pH meter (CP—411, Elmetron, Zabrze, Poland) with a glass penetrating electrode. Before the analysis, the pH meter was calibrated using standard phosphate buffers (pH 4.00 and 7.00). Between measurements, the electrode was rinsed thoroughly with distilled water.

### 2.3. Colour Measurement

The color of the raw fillet was assessed using a NR 20XE Precision Colorimeter (Shenzhen 3NH Technology Co., Ltd., Shenzhen, China) with φ20 mm extended aperture. L* (lightness), a* (redness), and b* (yellowness) were automatically obtained after a light shot was discharged perpendicularly to the inside surface of fillet. Measurements were performed in triplicate. On the basis of this, the whiteness index (WI) and chromaticity (C) parameters were calculated using the fallowing equations:

WI = 100 − [(100 − L)^2^ + a^2^ + b^2^]^0.5^, (3)

C = (a^2^ + b^2^)^0.5^. (4)

### 2.4. Chemical Analysis

Proximate analysis of samples (proteins, dry mater, lipids, ash) were determined according to AOAC (Association of Official Analytical Chemists) procedures [11]. Dry matter was calculated on the basis of moisture measurement. Moisture was obtained after drying samples in an oven at 105 °C for 24 h, whereas ash content was determined after incineration at 550 °C for 6 h. Crude protein was measured by determining nitrogen content (N × 6.25), according to the Kjeldahl method, using a Tecator Kjeltec 2100 distillation unit (FOSS Analytical Co., Ltd., Suzhou, China). Crude lipid was determined gravimetrically, after Soxhlet lipid extraction on a Tecator Soxtec System HT 1043 (FOSS Analytical Co., Ltd., Suzhou, China). Fatty acid profiles in meat samples (*n* = 50) were quantified using gas chromatography (GC) with a flame ionization detector (FID). Briefly, fatty acids were determined as fatty acid methyl esters (FAME), and individual FAME were identified by comparing their retention times with those of pure standards. Analyses were carried out in triplicate on an Agilent 6890N Network Gas Chromatograph (Agilent Technologies; Palo Alto, CA, USA) equipped with a 7683 automatic liquid sampler and flame ionization detectors. The amino acid profile of proteins (*n* = 20) in the abdomen and chelae meat samples was determined by High Performance Liquid Chromatography (HPLC) using an AAA 400 amino acid analyzer (Ingos, Prague, Czech Republic). The chromatograms were analyzed using the CHROMuLAN V 0.88 program (PiKRON, Prague, Czech Republic) by comparison with the standard chromatogram, taking the dilution and weight into account. All analyses were performed in triplicate.

### 2.5. Nutritional Value

Energetic value was calculated using the relative percentage of each nutrient (protein and fat), which was multiplied by the correction factors, 4 kcal g^−1^ (17 kJ g^−1^) and 9 kcal g^−1^ (37 kJ g^−1^) for protein and fat, respectively, as described in the EU Regulation No 1169/2011 [12].

Fat quality was described by the following factors: SFA (sum of saturated fatty acids), MUFA (sum of monounsaturated fatty acids), PUFA (sum of polyunsaturated fatty acids), h:H (hypocholesterolemic/hypercholesterolemic ratio), [13], IA (index of atherogenicity), [14,15] and IT (index of thrombogenicity), [15]. These factors were calculated using the fallowing equations:

SFA = (C12:0 + C14:0 + C15:0 + C16:0 + C17:0 + C18:0 + C20:0 + C22:0 + C24:0),(5)

MUFA = (C16:1n7 + C17:1n7 + C18:1n9t + C18:1n9c + C20:1n5 + C20:1n9 + C22:1n9 + C24:1n9),(6)

PUFA = (C18:2n6t + C18:2n6c + C18:3n6 + C18:3n3 + C20:2n6 + C20:3n3 + C20:3n6 + C20:4n6 + C20:5n3 + C22:2n6 + C22:6n3),(7)

h:H = Σ(C18:1n9, C18:1n7, C18:2n6, C18:3n6, C18:3n3, C20:3n6,C20:4n6, C20:5n3, C22:4n 6, C22:5n3, C22:6n3)/Σ(C14:0, C16:0), (8)

IA = (C12:0 + 4 × C14:0 + C16:0)/((n − 6)PUFA + (n − 3)PUFA + MUFA), (9)

IT = (C14:0 + C16:0 + C18:0)/(0.5 × MUFA + 0.5 × (n − 6)PUFA + 3.0 × (n − 3)PUFA + (n − 3)PUFA/(n − 6)PUFA).(10)

### 2.6. Structure

From each fillet, samples of 5 × 5 × 10 mm were cut, fixed for 12 h in Sannomiya solution, dehydrated using alcohol saturated in intermediary solutions (benzene, benzene:paraffin), embedded in paraffin blocks, and sectioned with the Rotary Microtome MPS-2 (Opta-Tech, Warsaw, Poland) into with 10 ± 1 μm slices. The sections were mounted onto clear glass slides, contrast-stained with hematoxylin and eosin, and sealed with Canada balsam [16]. MultiScanBase v.13.01 (Computer Scanning System Ltd., Warsaw, Poland), a computer image analysis software, was used to measure muscle fiber parameters, i.e., cross-sectional area (CSA), and horizontal (H) and vertical (V) diameters, as well as the thickness of connective tissue surrounding every muscle fiber (endomysium). For each excised sample, 3 slides were prepared and analyzed. Approximately 150 muscle fibers and up to 100 endomysium were measured in each slide. Values of H were divided by V to calculate the shape coefficient of muscle fiber. Its values fell within the 0 and 1 range. It was assumed that the closer the coefficient was to 1, the more regular the shape of the fibers would be.

### 2.7. Cooking Loss

In the next step, right fillets were weighted and steamed under atmospheric pressure in a steamer until temperature reached 68 ± 1 °C in the thickest part of fillet, and then cooled to 5 ± 1 °C. Temperature in the middle of the samples was measured with a thermometer (DT—34, Termoprodukt, Bielawa, Poland). After chilling, the samples were weighted again to calculate cooking loss.

### 2.8. Texture Profile Analysis

The texture of steamed fillets was measured with an Instron 1140 machine (Stable Instron, Bucks, UK), applying a double penetration TPA test [17]. In the TPA test, a 9.6 mm shaft was driven twice into the sample for a depth of 16 mm (80% deformation) and parameters such as hardness (N), cohesiveness (-), springiness (cm), and chewiness (N × cm) were measured. Crosshead speed was 50 mm/min. The device was calibrated each time before starting the analyses. The TPA test was applied a minimum of five times for each fillet.

### 2.9. Sensory Analysis

Sensory evaluation of steamed fillets was conducted by a trained team, consisting of four members, reaching sensory minimum [18]. All respondents have consented to participation in the study. Texture characteristics (tenderness, juiciness, perceptibility of connective tissue, chewiness), fattiness, gaping, odor, and taste intensity (fishy and geosmine) were evaluated. Each feature intensity was rated using a 5-point scale, where 1 point corresponded to the lowest, and 5 points corresponded to the highest feature intensity. Connective tissue perceptibility was regarded as the amount and degree of connective tissue perception located between muscle fiber bundles, responsible for the samples’ cohesiveness. Gaping was assessed as the easiness of separating the myomeres.

### 2.10. Statistical Analysis

The data were analyzed using STATISTICA for Windows (version 13.1, Cracow, Poland). The significance of differences between the samples was assessed using Tukey’s honestly significant difference test at the probability level of *p* = 0.05. The effects of biological factors (fish group, sex) were subjected to a multiple analysis of variance (ANOVA) at the significance level of 0.05 and 0.01.

## 3. Results

### 3.1. Zootechnical Parameters

The results of the zootechnical parameters of fish are presented in Table 1. There were no significant (*p* > 0.05) differences in the weight of body (BW), carcass (CW), and fillets (FW) between the groups of fish studied. However, the greater CW and FW heteroclarias resulted in a significantly (*p* ≤ 0.05) higher carcass (by approx. 13%) and fillet (by approx. 10%) yield compared to *C. gariepinus*.

The sex of the fish had no effect on any of the biometric traits tested, although females had higher fillet weight (FW), and males had higher weight (BW) and carcass yield.

### 3.2. Proximate Composition and Fat Characteristics of Fillets

There was no effect of fish group on protein content, dry matter, and fat in fillets, and their energy value (Table 2). Compared to *C. gariepinus*, heteroclarias fillets had a significantly (*p* ≤ 0.01) higher level of ash (by approx. 7%) and insignificantly (*p* > 0.05) higher content of protein and dry matter, and a lower level of fat and energy value.

The sex of the fish had a significant effect on the protein and fat content in the muscles and meat energy value. Female fillets had significantly (*p* ≤ 0.01) higher protein and fat content (by approx. 11% and 92%, respectively) and higher energy value (by approx. 32–34%), and an insignificantly (*p* > 0.05) higher level of dry matter compared to males.

The fatty acids (FA) profile analysis of fish fat (Table S1) showed that palmitic acid (C16:0) was the most abundant saturated fatty acid (SFA); the sum of oleic (C18:1n9c) and elaidic (C18:1n9t) acids was the most abundant monounsaturated fatty acid (MUFA); and linoleic acid (C18:2n6c) was the most abundant polyunsaturated fatty acid (PUFA). Muscle fat of heteroclarias contained more C16:0 and C18:1n9c+ C18:1n9t, and lower content of C18:2n6c than fat of Clarias. The muscle fat of females had a higher content of palmitic acid, sum of oleic and elaidic acids, and lower content of linoleic acid in comparison to the muscle fat of males. Comparing the fat quality of *C. gariepinus* and heteroclarias, significant (*p* ≤ 0.05) differences were found between fish groups (Table 3).

Heteroclarias fat was characterized by a higher content of SFA and a lower content of MUFA and PUFA, a lower amount of FA from the n − 6 and n − 9, and a lower ratio of n − 6:n − 3 and PUFA:SFA. Fish group had no effect on n − 3 amount, but in heteroclarias fat, we found a significantly (*p* ≤ 0.01) higher content of EPA and insignificantly (*p* > 0.05) lower content of DHA. *C. gariepinus* fat was characterized by significantly (*p* ≤ 0.01) lower IA and IT indices and a higher h:H ratio than heteroclarias fat.

The sex of fish had a significant effect (*p* ≤ 0.01) on the quality of muscle fat. Female fat compared to male had a significantly (*p* ≤ 0.01) higher content of SFA and a significantly (*p* ≤ 0.01) lower content of PUFA, n – 3, and n − 6 fatty acids (Table 3). A significantly (*p* ≤ 0.01) higher amount of EPA was found in female fat, and a higher amount of DHA was found in male fat. Female fat was characterized by significantly (*p* ≤ 0.01) lower PUFA:SFA ratio and h:H ratio, and significantly (*p* ≤ 0.01) higher IA and IT indices.

### 3.3. Color of Fillets

**Figure 1:**
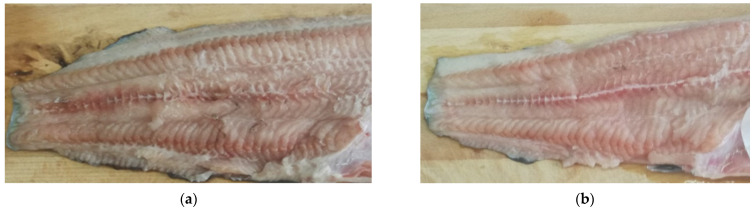
Color of fillets: (**a**) *C. gariepinus*, (**b**) heteroclarias.

No significant (*p* > 0.05) differences in color components (L*, a*, b*) and brightness coefficient (WI) between fish groups were found (Table 4). Fish groups differed significantly (*p* ≤ 0.05) only in color saturation (parameter C). However, it can be seen that the heteroclarias were lighter in color than *C. gariepinus* (Figure 1), as evidenced by the greater brightness (L*) and whiteness ratio (WI), as well as lower a* and b* parameters noted in the fillets of the hybrids.

The sex of the fish had a significant (*p* ≤ 0.01) effect on the brightness (L*) and redness (a*) of the color, as well as on the calculated brightness coefficients (WI) and color saturation (C), (*p* ≤ 0.05). Female fillets were lighter in color (about 10% higher by the L* parameter and about 11% by the WI), and about 20% less red (a*) and with about 15% less color saturation (C) than male meat.

### 3.4. pH and Cooking Loss of Fillets

A highly significant (*p* ≤ 0.01) effect of fish group on the pH of meat was demonstrated (Table 4). Heteroclarias meat was characterized by about 6% higher pH than *C. gariepinus* meat, and as a consequence, it also had smaller (*p* > 0.05) mass losses after heat treatment. No significant (*p* > 0.05) effect of fish sex on the pH value of their meat and the amount of cooking losses was demonstrated.

### 3.5. Muscle Structure

Fish group significantly (*p* ≤ 0.01) differentiated the size of muscle fibers, but had no effect (*p* > 0.05) on the shape of the fibers and the thickness of the endomysium (Table 5). Heteroclarias muscle fibers had about 8% greater average cross-sectional area compared to *C. gariepinus*. The hybrid endomysium was slightly thinner than the connective tissue of *C. gariepinus*.

Fish sex had a significant (*p* ≤ 0.01) effect on the size and shape of muscle fibers. Female muscle fibers compared to male ones were characterized by about 23% larger cross-sectional area and less regular shape. There was no significant (*p* > 0.05) effect of fish sex on the thickness of connective tissue in their muscles, although thicker endomysium was observed in female muscles.

### 3.6. Texture of Fillets

By analyzing the fillet texture (Table 6), it was shown that the fish group only significantly affected (*p* ≤ 0.05) fillet hardness. Heteroclarias meat was characterized by a significantly (*p* ≤ 0.05) higher (by approx. 30%) hardness and an insignificantly (*p* > 0.05) higher cohesiveness and chewiness, as well as an insignificantly (*p*> 0.05) lower springiness.

The sex of the fish did not affect the TPA test parameters. However, female meat was insignificantly (*p* > 0.05) more tough, cohesive, and springy, and consequently, also more chewy than male meat.

### 3.7. Sensory Assessment of Fillets

The study showed no significant (*p* > 0.05) differences in sensory quality between the fish groups (Table 7). However, it can be noted that heteroclarias fillets have been rated as less tender, juicy, fatty, and chewy than catfish fillets. They also noted lower perceptibility of connective tissue, gaping, and less intense taste and smell of fish and geosmine.

Fish sex was a factor that differentiated connective tissue perceptibility and chewiness of meat. Female fillets had significantly (*p* ≤ 0.01) lower perceptibility of connective tissue and were significantly (*p* ≤ 0.05) easier to chew than male fillets. At the same time, female fillets had insignificantly (*p* > 0.05) lower tenderness, juiciness, fattiness, and gaping, and with a lower intensity of fish and geosmine flavors, and a higher intensity of fishy and geosmine odors.

## 4. Discussion

Hybrid production has its economic justification. In aquaculture, it allows for better culture parameters, i.e., higher growth rates, fillet yield, and lower mortality due to diseases. The study showed that the studied fish groups did not differ significantly in body, carcass, and fillet weight, but a significantly higher carcass and fillet yield was shown for heteroclarias (68.3 and 53.9%, respectively) compared to *C. gariepinus* (60.4 and 49.1%). According to the literature data, the yields of *C. gariepinus* carcasses and fillets were at the level of 66.75–69.95% and 42.69%, respectively [5,19], and were similar to those found in our study, whereas the higher dressing percentage of heteroclarias confirms its higher utility value compared to *C. gariepinus*.

The consumer acceptance of raw materials and fish products depends on, among other things, their nutritional value and sensory properties, especially color, tastiness, and texture [20,21]. In turn, the chemical usefulness of the raw material is determined by its chemical composition, losses during storage and thermal treatment, as well as the pH of the meat. Our study showed, as well as the literature data, that African catfish meat has a good nutritional value [22,23,24], thus it can replace raw materials and animal products in the human diet. Additionally, the results did not show significant differences in the basic chemical composition or the energy values of heteroclarias and *C. gariepinus* meat. Toko et al. [25] did not show significant differences in the protein content between the meat of African catfish and *H. longifilis*; however, they recorded a higher fat content in *H. longifilis* meat, which was particularly evident at high stocking density. We found about 17% protein and 4% fat in *C. gariepinus* meat, which means that fish of this species can be classified as medium-fat [26]. This was also confirmed by the results obtained for this species (16.8–17.42% of protein, and 5.3–5.7% of fat) by Rosa et al. [22], Chwastowska-Siwiecka et al. [5], and Chwastowska-Siwiecka et al. [27]. However, the content of protein (17%) and fat (3%, low-fat species) in the heteroclarias meat was respectively higher and lower than that observed by Olaniyi et al. [24] in the meat of *H. bidorsalis* (66.8% crude protein—approx. 14.9% protein in d.m., and 21.5% ether extract—approx. 4.8% lipids in d.m.). In addition to protein and fat, the nutritional value of the raw material is also determined by the quality of the fat (fatty acid profile). Our study showed that the fat of *C. gariepinus* and heteroclarias fillets had a high content of MUFA (48.4–49.9%) and similar share of SFA (22.2–25.9%) and PUFA (25.6–27.8%), but the fat of the hybrid fillet was more saturated and had a lower content of n − 6 and n − 9 fatty acids. In both of the fish groups, the fat was characterized by a comparable amount of n − 3 FA (including the sum of EPA and DHA), but the fat of heteroclarias had significantly higher DHA content. This essential unsaturated fatty acid is responsible for reducing the risk of cancer and cardiovascular disease [28,29,30], and is particularly beneficial in the nutrition of pregnant women and children due to its beneficial effect on development and the nervous system, among others [31]. A similar share of three FA fractions (SFA, MUFA, PUFA) in African catfish meat was shown by Abouel-Yazeed [32]. According to Rosa et al. [22], it was found that the content of SFA and PUFA in *C. gariepinus* was higher (approx. 32.7 and 36.3%, respectively), and MUFA was lower (approx. 30.9%) than that found in our work. These differences in protein and fat content and in the fatty acid profile may result from different fish farming conditions (feed composition) and fish weight in compared experiments. Rosa et al. [22] conducted research on fish weighing approx. 2052 g, whereas individuals in our study weighed approx. 1100 g, and as is also described in the literature, with the age (weight) of animals, the fat content increases, and the protein content in their body decreases [33], and the fatty acid profile changes [34]. On the other hand, the type of feed determines the content of protein and fat and its quality in the body of fish [34,35], especially the content and type of lipid component in feed [4,36,37,38]. The study showed that the fat of both fish groups has a high health value due to higher than recommended by nutritionists [39,40] ratios, PUFA:SFA > 0.45 (1.0–1.3 in our study), n − 6:n − 3 < 4 (2.7–3.1 in our study), index of atherogenicity IA < 1.0 (0.25–0.30 in our study), index of thrombogenicity IT < 0.5 (0.37–0.45 in our study), and high h:H ratio (3.64–4.42 in our study). These quality parameters of fat were found in catfish fillets. Similarly, the high nutritional value of catfish fat has been confirmed by Abouel-Yazeed [32].

In the sensory assessment, hybrid meat received better notes than African catfish, which, according to literature data, is characterized by high sensory attractiveness, including tenderness, which is a consequence of the low content of connective tissue proteins in the meat of this species [5], at a level of approx. 3% of total protein [41]. In our research, we showed that heteroclarias had a less tender, but easier to chew, meat, and therefore, it was better digested, especially compared to meat from slaughter animals [42]. We observed low-level gaping in both fish groups, as confirmed by the preserved fillet structure—clearly visible muscle fibers surrounded by endomysium. The gaping phenomenon is caused by damage to the fish meat structure at the fiber and myocommata attachments level, which causes the formation of slits or holes in the fillet surface [20]. Kiessling et al. [21] suggest that fish with many small fibers would have a relatively larger amount of connective tissue, which would prevent the fillet from gaping. Hence, smaller muscle fibers that have been observed in hybrid muscles may be responsible for the slightly smaller gaping of the heteroclarias fillets. On the other hand, smaller heteroclarias muscle fibers may also indicate a greater degree of packing of their muscle structure, which, in turn, resulted in significantly higher hardness and insignificantly higher chewiness of hybrid meat. Variation in muscle structures is an important determinant of texture and other flesh quality characteristics [20]. Fish with smaller average cross-sectional area have a higher sensory firmness [20,43,44]. A higher muscle fiber density was positively and significantly correlated with textural properties (hardness, springiness, cohesiveness, and chewiness) of sea bass (*Dicentrarchus labrax*) meat [45], as well as with a lower amount of fat and “oiliness score” in Atlantic salmon (*Salmo salar*) muscles [46], which is consistent with our results. However, meat texture does not depend exclusively on its structure properties. According to the literature [21,47], texture is strongly affected by the chemical composition of meat (amount protein, fat), as well as type, structure, and functional properties of proteins. Our study showed that the greater hardness of heteroclarias meat resulted from the higher content of protein, lower content of fat, and higher saturation of fat. Similarly, Saavedara et al. [38] found a correlation between a lower amount of fat in meat and higher hardness. Fish lipids and lipid-derived aroma compounds, which are produced by the enzymatic oxidation of the polyunsaturated fatty acids present in fish, are responsible for the typical fish taste and smell [47]. Although our study showed differences in the content of PUFA, no significant differences were found between palatability parameters of *C. gariepinus* and heteroclarias meat. The meat of fish from both groups was characterized by a low intensity of fish smell and taste, and a low intensity of taste deviations (geosmine taste), which makes this raw material attractive to consumers who are reluctant to consume fish due to its taste and flavor. However, Olaniyi et al. [24] showed that hybrid meat in comparison to parental species (*C. gariepinus*, *H. bidorsalis*) had poorer sensory acceptability. These differences in sensory evaluation of hybrid meat in both experiments are probably the result of using other species of the catfish family, or another feed composition used in the nutrition for their production.

In the technological assessment of raw materials, parameters such as color, pH, and WHC (water holding capacity) are used. Compared to *C. gariepinus*, heteroclarias meat had a lighter color, as evidenced by greater brightness (L*) and whiteness index (WI), as well as lower a* and b* parameters. The color of meat is determined by, among others, its pH—lighter muscles have higher pH values than darker muscles [48]. We also found this relationship between pH and meat color in our work, whereas Kralik et al. [49] and Saláková et al. [50] determined a negative correlation between pH and L*. Meat pH is a parameter that determines weight loss during storage and processing, as well as the texture of the meat [41]. We have shown in studies that a higher pH of hybrid meat was responsible for higher hardness and chewiness, as well as lower cooking losses compared to African catfish meat. A similar relationship between pH and drip loss was obtained by Kralik et al. [44], and a relationship between pH and texture parameters was shown by Periago et al. [45].

When choosing and purchasing fish, consumers are guided mainly by their price and nutritional value, as well as by species. They do not take into account the sex of the fish because they are not able to assess whether the fish, which they most often buy in the form of a fillet, is male or female. In our research, we showed that sex significantly affected the amount of protein and fat in the meat, as well as fat quality, meat color (L*, WI, a*, and c), size and shape of fibers, sensory perception of connective tissue and chewiness, and the energy value of the meat. However, this factor did not affect carcass and fillets yields, dry matter, ash and pH, cooking loss, endomysium thickness, TPA parameters, and most of the sensory traits. Moreover, [19] did not show the effect of *C. gariepinus* sex on the performance of edible parts (carcass and fillet yields), although in males, they found a significantly higher proportion of head and fins, whereas in females—guts (due to the greater weight of gonads). In turn, Chwastowska-Siwiecka et al. [27] showed significant differences between males and females in thermal loss, shear force value, and color parameters of their meat. Biró et al. [51] found significant differences between the two sexes in the n-3 PUFA, resulting in a higher n − 3/n − 6 ratio in the male fillets of Nile tilapia. Akpinar et al. [52] showed a significantly higher share of SFA, MUFA, and n − 3 PUFA (including EPA) in female fillets than male fillets of *Salmo trutta macrostigma*. The available literature lacks information on the effect of fish sex on the culinary and processing quality of their meat. This biological factor also determines the quality of raw fish material, and can probably be the reason for differences in the consumer assessment of the quality of fish fillets of the same species.

## 5. Conclusions

We concluded that heteroclarias meat can be an interesting “species” for both breeders and consumers. The production of heteroclarias (H) hybrids allows to increase the yields of carcasses and fillets, without compromising the quality of their meat compared to *Clarias gariepinus* (C). The meat of C and H fishes had similar proximal composition, but C had better fat quality, although EPA content was higher in H meat. Due to the beneficial composition of fat in catfish and heteroclarias meat, their presence in the human diet may contribute to reducing the risk of coronary heart disease and cancer. Slightly better culinary and processing quality was found for H fillets, which were characterized by a more delicate meat structure (smaller muscle fibers and thinner endomysium); more saturated, lighter, and less red color; higher pH and lower thermal drip losses; and obtaining better scores in the sensory evaluation of texture, taste, and smell than C fillets. For this reason, heteroclarias meat can be an attractive raw material for more demanding groups of consumers—for children and adolescents (due to less intense fish tastiness), as well as for older people who have problems with chewing and digesting food (due to lower sensory chewiness and perception of connective tissue). The sex of the fish did not differentiate carcass and fillet yields, texture, and most of the sensory parameters, but had a significant effect on quality of fat, the color, size of muscle fibers, as well as the perception of connective tissue and the chewiness of fish meat. The consequence of this may be differences in consumer meat quality assessment among the same fish species.

The results of this study will contribute to the development of global aquaculture and the food industry. However, further studies are necessary to develop sustainable feeding programs for catfish, to increase production efficiency, and to provide further characteristics of the nutritional quality of meat, especially protein quality and the content of elements.

## Figures and Tables

**Table 1:** Effect of fish group and sex on zootechnical traits. Values are expressed as mean ± standard deviation (SD).

	Fish Group (F)		Sex (S)		Significance of Influence		
Traits	*C. gariepinus*	Heteroclarias	Male	Female	F	S	F × S
BW (g)	1138 ± 156.7	1170 ± 76.3	1137 ± 149.1	1169 ± 89.9	n.s.	n.s.	n.s.
CW (g)	688 ± 109.9	763 ± 52.9	732 ± 97.5	719.1 ± 91.8	n.s.	n.s.	n.s.
FW (g)	280 ± 45.7	315 ± 28.6	293 ± 42.6	301.8 ± 42.1	n.s.	n.s.	n.s.
Carcass yield (%)	60.4 ^a^ ± 3.96	68.3 ^b^ ± 1.75	64.4 ± 1.98	61.3 ± 4.76	**	n.s.	*
Fillets yield (%)	49.1 ^a^ ± 2.92	53.9 ^b^ ± 1.27	51.6 ± 2.49	51.4 ± 4.49	*	n.s.	n.s.

^ab^—values in rows with different index differ significantly (*p* ≤ 0.05) between fish group; Significance of Influence: n.s.—non-significant, * *p* ≤ 0.05, ** *p* ≤ 0.01; Explanations: BW—fish body weight, CW—carcass weight, FW—fillets with skin weight.

**Table 2:** Effect of fish group and sex on proximate composition of fillets. Values are expressed as mean ± standard deviation (SD).

	Fish Group (F)		Sex (S)		Significance of Influence		
Component (% of Wet Weight)	*C. gariepinus*	Heteroclarias	Male	Female	F	S	F × S
Protein	16.8 ± 1.24	17.0 ± 1.37	16.0 ^A^ ± 0.04	17.8 ^B^ ± 0.18	n.s.	**	n.s.
Dry matter	22.4 ± 0.68	23.5 ± 1.59	22.2 ± 0.30	23.8 ± 1.22	n.s.	n.s.	n.s.
Fat	4.0 ± 2.31	3.0 ± 0.86	2.4 ^A^ ± 0.01	4.6 ^B^ ± 1.46	n.s.	*	n.s.
Ash	1.13 ^a^ ± 0.02	1.21 ^b^ ± 0.01	1.17 ± 0.07	1.17 ± 0.05	*	n.s.	n.s.
Energy value							
(kcal 100 g^−1^)	102.5 ± 27.11	94.7 ± 13.18	84.38 ^A^ ± 1.46	112.9 ^B^ ± 12.47	n.s.	*	n.s.
(kJ 100 g^−1^)	434.9 ± 106.33	398.9 ± 54.95	359.9 ^A^ ± 10.23	473.9 ^B^ ± 51.15	n.s.	*	n.s.

^ab^—values in rows with different index differ significantly (*p* ≤ 0.05) between fish group; ^AB^—values in rows with different index differ significantly (*p* ≤ 0.05) between sex; Significance of Influence: n.s.—non-significant, * *p* ≤ 0.05, ** *p* ≤ 0.01.

**Table 3:** Effect of fish group and sex on fat characteristics of fillets. Values are expressed as mean ± standard deviation (SD).

	Fish Group (F)		Sex (S)		Significance of Influence		
Traits	*C. gariepinus*	Heteroclarias	Male	Female	F	S	F × S
SFA (% FA in fat)	22.2 ^a^ ± 0.84	25.9 ^b^ ± 1.18	22.5 ^A^ ± 1.24	24.4 ^B^ ± 2.31	**	**	n.s.
MUFA (% FA in fat)	49.9 ^a^ ± 0.87	48.4 ^b^ ± 0.70	49.3 ± 1.03	49.5 ± 1.22	**	n.s.	n.s.
PUFA (% FA in fat)	27.8 ^a^ ± 0.53	25.6 ^b^ ± 1.11	28.1 ^A^ ± 0.25	26.0 ^B^ ± 1.53	**	**	**
n − 3 (% FA in fat)	6.92 ± 0.31	6.93 ± 0.43	7.14 ^A^ ± 0.16	6.68 ^B^ ± 0.03	n.s.	**	n.s.
EPA (% FA in fat)	0.70 ^a^ ± 0.03	0.73 ^b^ ± 0.01	0.69 ^A^ ± 0.03	0.73 ^B^ ± 0.01	**	**	**
DHA (% FA in fat)	2.68 ± 0.29	2.50 ± 0.37	2.87 ^A^ ± 0.04	2.34 ^B^ ± 0.24	n.s.	**	n.s.
EPA + DHA (% FA in fat)	3.38 ± 0.28	3.23 ± 0.37	3.56 ^A^ ± 0.06	3.07 ^B^ ± 0.25	n.s.	**	n.s.
EPA + DHA (g 100g ^−1^ ww)	0.110 ± 0.04	0.098 ± 0.01	0.085 ^A^ ± 0.001	0.130 ^B^ ± 0.04	n.s.	**	n.s.
n − 6 (% FA in fat)	20.87 ^a^ ± 0.36	18.77 ^b^ ± 1.39	20.91 ^A^ ± 0.40	19.32 ^B^ ± 1.47	**	**	**
n − 9 (% FA in fat)	47.76 ^a^ ± 0.84	46.17 ^b^ ± 0.67	47.21 ± 1.10	47.25 ± 1.16	**	n.s.	n.s.
PUFA:SFA	1.25 ^a^ ± 0.05	1.0 ^b^ ± 0.12	1.24 ^A^ ± 0.07	1.08 ^B^ ± 0.16	**	**	**
n − 6:n − 3	3.02 ^a^ ± 0.13	2.71 ^b^ ± 0.03	2.93 ± 0.12	2.90 ± 0.25	**	n.s.	n.s.
n − 3:n − 6	0.33 ^a^ ± 0.01	0.37 ^b^ ± 0.004	0.34 ± 0.01	0.34 ± 0.03	**	n.s.	n.s.
IA	0.25 ^a^ ± 0.01	0.30 ^b^ ± 0.01	0.25 ^A^ ± 0.02	0.28 ^B^ ± 0.03	**	**	n.s.
IT	0.37 ^a^ ± 0.02	0.45 ^b^ ± 0.04	0.38 ^A^ ± 0.02	0.43 ^B^ ± 0.05	**	**	**
h:H	4.42 ^a^ ± 0.21	3.64 ^b^ ± 0.19	4.35 ^A^ ± 0.31	3.94 ^B^ ± 0.46	**	**	n.s.

^ab^—values in rows with different index differ significantly (*p* ≤ 0.05) between fish group; ^AB^—values in rows with different index differ significantly (*p* ≤ 0.05) between sex; Significance of Influence: n.s.—non-significant, ** *p* ≤ 0.01; Explanations: SFA—saturated, MUFA—monounsaturated, PUFA—polyunsaturated fatty acids, FA—total fatty acids, ww—muscle wet weight.

**Table 4:** Effect of fish group and sex on color parameters, pH, and cooking loss of fillets. Values are expressed as mean ± standard deviation (SD).

	Fish Group (F)		Sex (S)		Significance of Influence		
Traits	*C. gariepinus*	Heteroclarias	Male	Female	F	S	F × S
L*	47.6 ± 1.57	49.3 ± 4.40	46.1 ^A^ ± 1.43	50.8 ^B^ ± 2.82	n.s.	**	n.s.
a*	12.0 ± 1.72	10.2 ± 1.43	12.3 ^A^ ± 1.26	9.9 ^B^ ± 1.32	n.s.	**	n.s.
b*	5.6 ± 0.71	5.1 ± 0.68	5.1 ± 0.78	5.7 ± 0.55	n.s.	n.s.	n.s.
WI	46.0 ± 1.65	48.0 ± 4.50	44.4 ^A^ ± 1.21	49.5 ^B^ ± 2.95	n.s.	**	n.s.
C	13.3 ^a^ ± 1.60	11.4 ^b^ ± 1.16	13.3 ^A^ ± 1.32	11.4 ^B^ ± 1.38	*	*	n.s.
pH	7.08 ^a^ ± 0.05	7.48 ^b^ ± 0.08	7.32 ± 0.25	7.27 ± 0.21	**	n.s.	n.s.
Cooking loss (%)	17.6 ± 1.59	15.7 ± 1.39	17.7 ± 1.47	15.6 ± 1.27	n.s.	n.s.	n.s.

^ab^—values in rows with different index differ significantly (*p* ≤ 0.05) between fish group; ^AB^—values in rows with different index differ significantly (*p* ≤ 0.05) between sex; Significance of Influence: n.s.—non-significant, * *p* ≤ 0.05, ** *p* ≤ 0.01.

**Table 5:** Effect of fish group and sex on structural elements of fillets. Values are expressed as mean ± standard deviation (SD).

	Fish Group (F)		Sex (S)		Significance of Influence		
Structure Element	*C. gariepinus*	Heteroclarias	Male	Female	F	S	F × S
Muscle fibre:							
CSA (µm^2^)	2148 ^a^ ± 203.1	1771 ^b^ ± 147.1	1758 ^A^ ± 122.9	2161 ^B^ ± 197.7	**	**	**
Shape (-)	0.90 ± 0.07	0.90 ± 0.06	0.92 ^A^ ± 0.02	0.87 ^B^ ± 0.02	n.s.	*	n.s.
Connective tissue (myocommata)							
*Endomysium* thickness (µm)	1.10 ± 0.16	1.04 ± 0.11	1.05 ± 0.11	1.09 ± 0.16	n.s.	n.s.	n.s.

^ab^—values in rows with different index differ significantly (*p* ≤ 0.05) between fish group; ^AB^—values in rows with different index differ significantly (*p* ≤ 0.05) between sex; Significance of Influence: n.s.—non-significant, * *p* ≤ 0.05, ** *p* ≤ 0.01.

**Table 6:** Effect of fish group and sex on TPA test parameters of fillets. Values are expressed as mean ± standard deviation (SD).

	Fish Group (F)		Sex (S)		Significance of Influence		
Parameter	*C. gariepinus*	Heteroclarias	Male	Female	F	S	F × S
Hardness (N)	1.50 ^a^ ± 0.13	1.95 ^b^ ± 0.25	1.71 ± 0.66	1.72 ± 0.45	*	n.s.	n.s.
Cohesiveness (-)	0.22 ± 0.09	0.23 ± 0.09	0.21 ± 0.08	0.24 ± 0.10	n.s.	n.s.	n.s.
Springiness (cm)	1.11 ± 0.19	1.02 ± 0.11	1.01 ± 0.14	1.11 ± 0.17	n.s.	n.s.	n.s.
Chewiness (N × cm)	0.39 ± 0.18	0.48 ± 0.25	0.30 ± 0.14	0.50 ± 0.22	n.s.	n.s.	n.s.

^ab^—values in rows with different index differ significantly (*p* ≤ 0.05) between fish group; Significance of Influence: n.s.—non-significant, * *p* ≤ 0.05.

**Table 7:** Effect of fish group and sex on sensory traits assessment of fillets. Values are expressed as mean ± standard deviation (SD).

	Fish Group (F)		Sex (S)		Significance of Influence		
Traits (pt.)	*C. gariepinus*	Heteroclarias	Male	Female	F	S	F × S
Tenderness	2.28 ± 0.73	2.03 ± 0.62	2.19 ± 0.68	2.13 ± 0.70	n.s.	n.s.	n.s.
Juiciness	2.19 ± 0.70	1.84 ± 0.60	2.09 ± 0.74	1.94 ± 0.60	n.s.	n.s.	n.s.
Perceptible of connective tissue	2.22 ± 0.58	2.09 ± 0.55	2.44 ^a^ ± 0.44	1.88 ^b^ ± 0.11	n.s.	**	n.s.
Chewiness	2.31 ± 0.57	2.28 ± 0.55	2.53 ^a^ ± 0.19	2.06 ^b^ ± 0.20	n.s.	*	n.s.
Fattiness	1.88 ± 0.74	1.63 ± 0.65	1.78 ± 0.73	1.72 ± 0.68	n.s.	n.s.	n.s.
Gaping	1.88 ± 0.70	1.59 ± 0.42	1.84 ± 0.68	1.63 ± 0.47	n.s.	n.s.	n.s.
Fish odour	1.75 ± 0.68	1.72 ± 0.48	1.72 ± 0.68	1.75 ± 0.48	n.s.	n.s.	n.s.
Geosmine odour	1.09 ± 0.27	1.09 ± 0.20	1.06 ± 0.17	1.13 ± 0.29	n.s.	n.s.	n.s.
Fish taste	2.19 ± 0.66	1.97 ± 0.50	2.19 ± 0.60	1.97 ± 0.56	n.s.	n.s.	n.s.
Geosmine taste	1.28 ± 0.45	1.22 ± 0.31	1.34 ± 0.44	1.16 ± 0.30	n.s.	n.s.	n.s.

^ab^—values in rows with different index differ significantly (*p* ≤ 0.05) between sex; Significance of Influence: n.s.—non-significant, * *p* ≤ 0.05, ** *p* ≤ 0.01.

## Data Availability

Data is contained within the article or Supplementary Material.

## Supplementary Materials

The following supporting information can be downloaded at: https://www.mdpi.com/article/10.3390/foods11142074/s1, Table S1. Fatty acid composition (% of FA in fat) of *C. gariepinus* and heteroclarias fat.
