# Supplementary material for: Does Production of Clarias gariepinus × Heterobranchus longifilis Hybrids Influence Quality Attributes of Fillets?
Source: Foods. 2022 Jul 12;11(14):2074. doi: 10.3390/foods11142074 (PMC9323170; doi:10.3390/foods11142074)
Supplement: Supplementary file 1 [file foods-11-02074-s001.zip › foods-1794113-supplementary.pdf]

Table S1. Fatty acid composition (% of FA in fat) of *C. gariepinus* and heteroclarias fat. Values are expressed as mean ± standard deviation (SD).

	C.gariepinus		Heteroclarias	
	Male	Female	Male	Female
(C12:0) Lauric acid	0.08 ± 0.01	0.10 ± 0.0001	0.09 ± 0.0001	0.07 ± 0.0
(C14:0) Myristic acid	0.94 ± 0.02	1.09 ± 0.02	1.06 ± 0.01	1.12 ± 0.01
(C15:0) Pentadecanoic acid	0.16 ± 0.004	0.17 ± 0.01	0.15 ± 0.01	0.15 ± 0.01
(C16:0) Palmitic acid	15.25 ± 0.05	15.33 ± 0.07	17.22 ± 0.05	18.37 ± 0.04
(C16:1n7) Palmitoleic acid	1.85 ± 0.02	2.09 ± 0.01	2.00 ± 0.01	2.01 ± 0.01
(C17:0) Heptadecanoic acid	0.24 ± 0.01	0.24 ± 0.01	0.21 ± 0.0001	0.21 ± 0.01
(C17:1n7) cis-10-Heptadecenoic acid	< LOD	0.05 ± 0.0001	0.05 ± 0.01	< LOD
(C18:0) Stearic acid	4.46 ± 0.02	4.44 ± 0.01	5.19 ± 0.01	6.17 ± 0.0001
(C18:1n9c + C18:1n9t) Oleic acid + Elaidic acid	45.66 ±0.13	46.20 ± 0.02	43.44 ± 0.06	44.62 ± 0.03
(C18:2n6c) Linoleic acid	17.70 ± 0.10	17.01 ± 0.02	17.19 ± 0.06	15.09 ± 0.02
(C18:2n6t) Linolelaidic acid	< LOD	< LOD	< LOD	0.06 ± 0.01
(C18:3n6) gamma-Linolenic acid [GLA]	1.37 ± 0.01	1.73 ± 0.01	0.94 ± 0.0001	0.73 ± 0.01
(C18:3n3) alfa- Linolenic acid [ALA]	3.39 ±0.03	3.39 ± 0.0001	3.60 ± 0.01	3.51 ± 0.01
(C20:0) Arachidic acid	0.41 ± 0.01	0.38 ± 0.01	0.36 ± 0.01	0.34 ± 0.0001
(C20:1n15) cis-5- Eicosenoic acid	0.17 ± 0.01	0.18 ± 0.01	0.15 ± 0.01	0.13 ± 0.01
(C20:1n9) cis-11-Eicosenoic acid	1.78 ± 0.03	1.81 ± 0.01	1.71 ± 0.0001	1.72 ± 0.01
(C20:2n6) cis-11.14-Eicosenoic acid	0.50 ± 0.01	0.50 ± 0.01	0.52 ± 0.01	0.53 ± 0.01
(C20:3n6) cis-8.11.14-Eicosatrienoic acid [DGLA]	0.89 ± 0.0041	0.77 ± 0.0001	0.82 ± 0.0001	0.74 ± 0.0001
(C20:4n6) Arachidonic acid [AA]	0.62 ± 0.0041	0.55 ± 0.0001	0.64 ± 0.01	0.57 ± 0.0001
(C20:3n3) cis-11.14.17-Eicosatrienoic acid[ETE]	0.14 ± 0.01	0.13 ± 0.01	0.16 ± 0.01	0.14 ± 0.01
(C20:5n3) cis-5.8.11.14.17-Eicosappentaenoic acid [**EPA**]	0.68 ± 0.004	0.72 ± 0.01	0.74 ± 0.01	0.73 ± 0.0001
(C22:0) Behenic acid	0.24 ± 0.01	0.22 ± 0.01	0.19 ± 0.0001	0.17 ± 0.01
(C22:1n9) Erucic acid	0.20 ± 0.0001	0.23 ± 0.10	0.17 ± 0.01	0.18 ± 0.0001
(C22:2n6) cis-13.16-Docosadienoic acid	< LOD	< LOD	0.13 ± 0.03	0.05 ± 0.03
(C24:0) Lignoceric acid	0.10 ± 0.01	0.09 ± 0.0001	0.008 ± 0.01	0.08 ± 0.0001
(C22:6n3) cis-4.7.10.13.16.19-Docosahexaenoic acid [**DHA**]	2.86 ± 0.04	2.26 ± 0.03	2.90 ± 0.01	2.23 ± 0.01
(C24:1n9) Nervonic acid	0.17 ± 0.01	0.17 ± 0.01	0.15 ± 0.01	0.14 ± 0.01

LOD – level of detection.
